# Mechanisms of quality control differ in male and female germ cells

**DOI:** 10.1038/s41418-021-00818-8

**Published:** 2021-06-15

**Authors:** Eleonora Candi, Gerry Melino, Attila Tóth, Volker Dötsch

**Affiliations:** 1 Department of Experimental Medicine, University of Rome “Tor Vergata”, Rome, Italy; 2 IDI-IRCCS, Via dei Monti di Creta, Rome, Italy; 3 Institute of Physiological Chemistry, Faculty of Medicine, Technische Universität Dresden, Dresden, Germany; 4 Institute of Biophysical Chemistry and Center for Biomolecular Magnetic Resonance, Goethe University, Frankfurt/Main, Germany

**Keywords:** Cell biology, Stem-cell research

Despite the high importance of protecting the genome in the germline, paradoxically, hundreds of DNA double strand breaks (DSBs) are generated by SPO11, in meiotic germ cells. These DSBs serve an important function as they initiate meiotic homologous recombination which is not only necessary to repair the DSBs, but also to promote the pairing of homologous chromosomes. At least one repair event per chromosome pair creates crossovers which is required for reliable chromosome segregation during cell division. Given that efficient repair of high numbers of DSBs is not straightforward, some of the meiotic germ cells fail to repair all of the SPO11-induced DSBs. Oocytes with persistent DSBs are eliminated by apoptosis before entering dictyate arrest, which is a resting stage of oocytes that can last up to 50 years in humans. The oocyte surveillance system that triggers cell death in response to DSBs has been characterized in recent years. It involves a member of the p53 protein family, TAp63α, that is preferentially produced in resting oocytes [1]. This p63 isoform adopts an inactive and only dimeric conformation [2]. ATM- and/or ATR-mediated detection of DSBs activates the DNA damage response kinase CHK2, which phosphorylates TAp63α at S582 [3]. While this phosphorylation is not sufficient to activate TAp63α, it recruits the kinase CK1 [4]. Sequential addition of four more phosphate groups results in activation of TAp63α to the tetrameric state [5] and initiation of apoptosis [6].

While many molecular details of this p63-mediated quality control mechanism are well-understood in oocytes, TAp63α’s function in the male germ line has remained poorly explored. Comparison of the transcriptomes of wild type and p63^+/-^ mouse germ cells suggested that p63 is involved in regulating an apoptotic program [7]. Elevated p53 and TAp63 levels also were observed in spermatocytes of *Trip13*^mod/mod^ mice, where DSB repair was delayed and spermatocytes were eliminated in mid prophase due to defective meiotic recombination and misregulation of HORMAD1/2 proteins that activate meiotic prophase checkpoints [8–10]. Interestingly, whereas TAp63-deficiency did not prevent apoptosis in *Trip13*^mod/mod^ spermatocytes, TAp63-deficiency enabled the expression of a late meiotic marker that is normally repressed in *Trip13*^mod/mod^ spermatocytes. This suggests that the quality control of spermatocytes involves TAp63, albeit the role of TAp63 seems to be redundant and/or limited in male meiosis [11]. Interestingly, male germ cells of humans and great apes express a modified form of TAp63α, GTAp63α [12], which has a 37 amino acid N-terminal extension that is thought to inhibit p63’s function [13]. This extension corresponds to a LTR of the human endogenous retrovirus 9 (ERV9). DNA damage triggers activation of GTAp63α which initiates apoptosis [12].

**Fig. 1:**
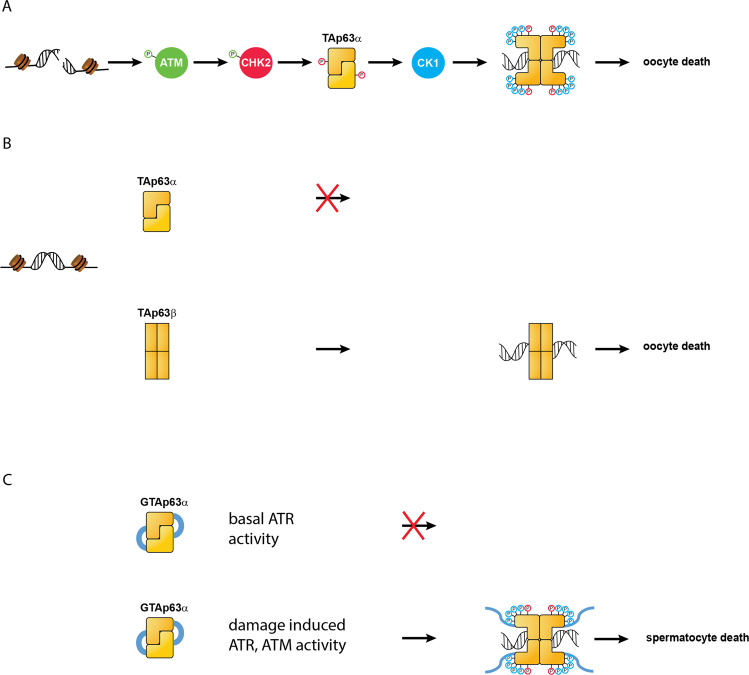
Activation of TAp63 in primary oocytes. **A** Unrepaired DNA double strand breaks initiate a kinase cascade that culminates in the phosphorylation of TAp63α (in its inactive, dimeric conformation) by CHK2. CHK2 phosphorylation permits the recruitment of another kinase, CK1, which adds four more phosphate groups. Phosphorylation by CK1 triggers a conformational change that results in an active and tetrameric state initiating oocyte death. **B** In a mouse model expressing both a full length TAp63α and a truncated TAp63β allele oocyte death is triggered by the constitutively tetrameric and active TAp63β form even without DNA damage. In contrast, in male germ cells of the same heterogeneous mouse model cell death is not initiated. **C** Speculative mechanism of quality control in male germ cells of humans and great apes. In these cells the GTAp63α isoform is expressed which is characterized by a 37 amino acid N-terminal extension. This extension (depicted with blue symbols) stabilizes the inactive conformation, potentially preventing activation in the presence of an elevated basal activity of the kinase ATR caused by the limited sex chromosome synapsis. In the presence of higher ART and/or ATM activity, caused for example by DSBs, activation could proceed via the normal Chk2/Chk1/CK1 route.

Whereas these listed observations suggest a similar role of p63 in male and in female germ cells, our recent mouse study cast doubts on p63’s importance for the quality control of spermatocytes. We created a heterozygous mouse expressing one wild type TAp63α allele and one TAp63β allele [14]. TAp63β is characterized by a shorter C-terminus that lacks the inhibitory domain, resulting in constitutively tetrameric and active p63 [15]. Consequently, female heterozygous mice had lost all oocytes by P7 even in the absence of any DNA damage. This result is consistent with the observation that p63 mutations that result in truncated proteins lacking its C-terminal inhibitory domain cause premature ovarian insufficiency in female patients [14]. Surprisingly, male heterozygous mice are fertile. If TAp63α activation promoted germ cell elimination in both male and female germ cells, then the constitutively active TAp63β form would be expected to trigger germ cell apoptosis in both sexes. The observed fertility in the TAp63α/TAp63β mouse model suggests that p63 is differentially regulated in male and female germ cells. These results might reflect the different developmental schemes of female and male germ cells that require distinct types of quality control mechanisms (Fig. 1). While oocytes are limited in number, divide in a highly asymmetric manner with only one daughter cell surviving and are arrested for years in a tretraploid state in prophase I, spermatocytes are constantly produced in high quantities, divide symmetrically and stay in prophase only for hours. Mechanistically, a potential explanation for sexual differences of p63’s role is rooted in differences between sex chromosomes in females and males, and in particular, the limited synapsis of the X and Y sex chromosomes in spermatocytes. Unsynapsed chromosomal regions promote ATR activity leading to the transcriptional silencing of unsynapsed sex chromosomes, which is essential for spermatocyte survival beyond meiotic prophase [10, 16]. It follows that ATR is constitutively active throughout meiotic prophase even in normal spermatocytes. Recombination defects and persistent DSBs on autosomes disrupt efficient sex chromosome silencing, which leads to spermatocyte apoptosis independent of p53 or p63 [11, 16]. Given these circumstances, the higher base level of ATR activity might make it necessary to keep TAp63α at low levels or not expressed at all. It is also possible that TAp63α activation occurs but does not contribute to apoptotic pathways in spermatocytes. Importantly, p63 loss enabled advancement in the meiotic transcriptional program, but p63 loss did not prevent apoptosis in DSB repair-defective *Trip13*^*mod/mod*^ spermatocytes [11]. Together with the TAp63α/TAp63β phenotype these observations suggest that male quality control in mice does not rely on p63-dependent apoptotic pathways and other factors possibly play decisive roles in spermatocytes. The situation in humans, however, might be different as the isoform that is selectively expressed in male germ cells of humans and great apes, GTAp63α [12], forms more stable dimers [13]. The N-terminal domain of the human protein might stabilize the inactive state on the background of a higher basal activity of the ATR kinase, which, counterintuitively, might allow the use of human GTAp63α for apoptosis induction if excess ATR/ATM signaling is present from damaged DNA. Clearly more research on the role of the different p63 isoforms in the male germ line is necessary to understand its role in quality control.
